# Anti-inflammatory and anti-amyloidogenic effects of a small molecule, 2,4-bis(p-hydroxyphenyl)-2-butenal in Tg2576 Alzheimer’s disease mice model

**DOI:** 10.1186/1742-2094-10-2

**Published:** 2013-01-05

**Authors:** Peng Jin, Jin-A Kim, Dong-Young Choi, Young-Jung Lee, Heon Sang Jung, Jin Tae Hong

**Affiliations:** 1 College of Pharmacy, Chungbuk National University, 12 Gaesin-dong, Heungduk-gu, Cheongju, Chungbuk, 361-763, Korea; 2 Medical Research Center, Chungbuk National University, 12 Gaesin-dong, Heungduk-gu, Cheongju, Chungbuk, 361-763, Korea; 3 College of Agriculture, Life and Environments Sciences, Chungbuk National University, 12, Gaeshin-dong, Heungduk-gu, Cheongju, Chungbuk, 361-763, Korea; 4 College of Pharmacy, Yeungnam University, Gyeongsan, Gyeongbuk, 712-749, Republic of Korea; 5 School of Equine industries, Cheju Halla University, 38 Halladaehak-ro, Jeju, 690-708, Korea

**Keywords:** Alzheimer’s disease, Amyloid-beta, NF-κB, STAT1/3, 2,4-bis(p-hydroxyphenyl)-2-butenal

## Abstract

**Background:**

Alzheimer’s disease (AD) is pathologically characterized by excessive accumulation of amyloid-beta (Aβ) fibrils within the brain and activation of astrocytes and microglial cells. In this study, we examined anti-inflammatory and anti-amyloidogenic effects of 2,4-bis(p-hydroxyphenyl)-2-butenal (HPB242), an anti-inflammatory compound produced by the tyrosine-fructose Maillard reaction.

**Methods:**

12-month-old Tg2576 mice were treated with HPB242 (5 mg/kg) for 1 month and then cognitive function was assessed by the Morris water maze test and passive avoidance test. In addition, western blot analysis, Gel electromobility shift assay, immunostaining, immunofluorescence staining, ELISA and enzyme activity assays were used to examine the degree of Aβ deposition in the brains of Tg2576 mice. The Morris water maze task was analyzed using two-way ANOVA with repeated measures. Otherwise were analyzed by one-way ANOVA followed by Dunnett’s post hoc test.

**Results:**

Treatment of HPB242 (5 mg/kg for 1 month) significantly attenuated cognitive impairments in Tg2576 transgenic mice. HPB242 also prevented amyloidogenesis in Tg2576 transgenic mice brains. This can be evidenced by Aβ accumulation, BACE1, APP and C99 expression and β-secretase activity. In addition, HPB242 suppresses the expression of inducible nitric oxide synthase (iNOS) and cyclooxygenase-2 (COX-2) as well as activation of astrocytes and microglial cells. Furthermore, activation of nuclear factor-kappaB (NF-κB) and signal transducer and activator of transcription 1/3 (STAT1/3) in the brain was potently inhibited by HPB242.

**Conclusions:**

Thus, these results suggest that HPB242 might be useful to intervene in development or progression of neurodegeneration in AD through its anti-inflammatory and anti-amyloidogenic effects.

## Background

Alzheimer’s disease (AD) is a fatal progressive neurodegenerative illness and the most common form of dementia [1]. AD is a devastating dementia that first presents as progressive memory loss and later can include neuropsychiatric symptoms [2]. Amyloidogenic processing of amyloid precursor protein (APP) by β- and γ-secretases leads to the production of Aβ peptides that can oligomerize and aggregate into amyloid plaques, a characteristic hallmark of AD [3]. Although the exact cause of AD remains elusive, mounting evidence continues to support the involvement of neuroinflammation in the development of AD [4]. Neuropathological studies in the human brain have demonstrated that the activated glial cells excessively release pro-inflammatory mediators and cytokines, which in turn triggerneurodegenerative cascades via neuroinflammation [5,6]. Inflammatory reactions and mediators have been reported to augment APP expression and Aβ formation [7,8] and transcriptionally upregulate mRNA and protein levels and enzymatic activity of β-secretase, a key enzyme in the production of Aβ [9,10].

Astrocytes and microglia are the major type of glial cells in the central nervous system and activation of these cells are involved in all types of neurodegenerative processes, indicating prominent remodeling in AD [11,12]. Activated astrocytes expressing glial fibrillary acidic protein (GFAP) are closely associated with AD pathology, such as Tau tangles, neuritic plaques and amyloid depositions [13]. Furthermore, astrocytes with increased beta-secretase 1 (BACE1) expression have been found in the brain in AD [14]. Fibrillar Aβ can activate microglia, resulting in production of toxic and inflammatory mediators like hydrogen peroxide, nitric oxide, and cytokines [15]. Microglial cells are closely associated with nearly all compact deposits of the Aβ-protein found in the senile plaques of AD [16]. Microglial activation is also involved in neuroprotection in the early phase, but, subsequently, extensive and continuous activation of microglia results in the neuroinflammation and Aβ accumulation in AD pathology [17].

Nuclear factor-kappa B (NF-κB) is a redox transcription factor that is critical for regulation of inflammation and various autoimmune diseases [12]. NF-κB is localized in the cytoplasm by the inhibitor of κB (IκB). After IκB is phosphorylated and degraded, NF-κB is released and translocated from the cytosol to the nucleus, and binds to its cognate DNA binding sites leading to expression of inflammatory mediators [18]. Expression of genes for inflammatory elements such as inducible inducible nitric oxide synthase (iNOS) and cyclooxygenase-2 (COX-2), as well as cytokines, can be regulated by activation of NF-κB [10]. NF-κB activation was also found to promote neuronal resistance to Aβ toxicity [19]. NF-κB signaling increases BACE1 expression [20] and NF-κB inhibitors decrease Aβ production and β-secretase activity [21]. Recently, it was reported that inhibition of the NF-κB pathway could enhance α-secretases, but inhibit β-secretase activity, and thereby reduce the formation of Aβ [22]. Signal transducer and activator of transcription (STAT)1 and STAT3 are also significant regulators of neuroinflammation, Aβ generation [23] and cytokine-driven NF-κB-mediated Aβ gene expression [24]. It was reported that resveratrol prevented the pro-inflammatory properties of fibrillar Aβ on macrophages by potently inhibiting the effect of Aβ on IκB phosphorylation, STAT1 and STAT3 activation [25]. Endogenous BACE1 levels were decreased by overexpression of suppressor of cytokine signaling (SOCS), an endogenous negative regulator of STAT1 signaling [26], demonstrating that downregulation of STAT1 signaling suppresses BACE1 expression and Aβ generation in neurons [27]. STAT3 is another transcription factor that is typically associated with cytokine signaling during neuronal differentiation and inflammation [28]. The STAT3 patwhay is disrupted in neurodegeneration induced by amyloid-peptide [29]. The STAT3-mediated transcriptional control of BACE1 leads to amyloidogenic processing of APP and Aβ generation [23].

The Maillard reaction (MR) products such as glucose-tyrosine and xylose-arginine have antioxidant, antimutagenic, and antibacterial effects [30-32]. Our previous studies have shown the fructose-tyrosine MR product, HPB242, inhibits lipopolysaccharide (LPS)-elevated Aβ levels in cultured astrocytes and microglial BV-2 cells through attenuation of LPS-induced neuroinflammatory reactions [10]. Therefore, in the present study, we investigated whether HPB242 improves memory function and inhibits amyloidogenesis via the prevention of neuroinflammation in Tg2576 mice.

## Methods

### Animals

In the present study we used Tg2576 mice as a model of AD. These mice overexpress human APP with the Swedish double mutations (K670N, M671L) under the control of a hamster prion protein promoter [33]. The 12-month-old Tg2576 female mice used in the present study were purchased from Taconic Farms (Germantown, NY, USA) and were maintained and handled in accordance with a protocol approved by the Institutional Animal Care and Use Committee of Laboratory Animal Research Centre at Chungbuk National University (Approval no. CBNU-144-1001-01).

### Chemicals

**Figure 1:**
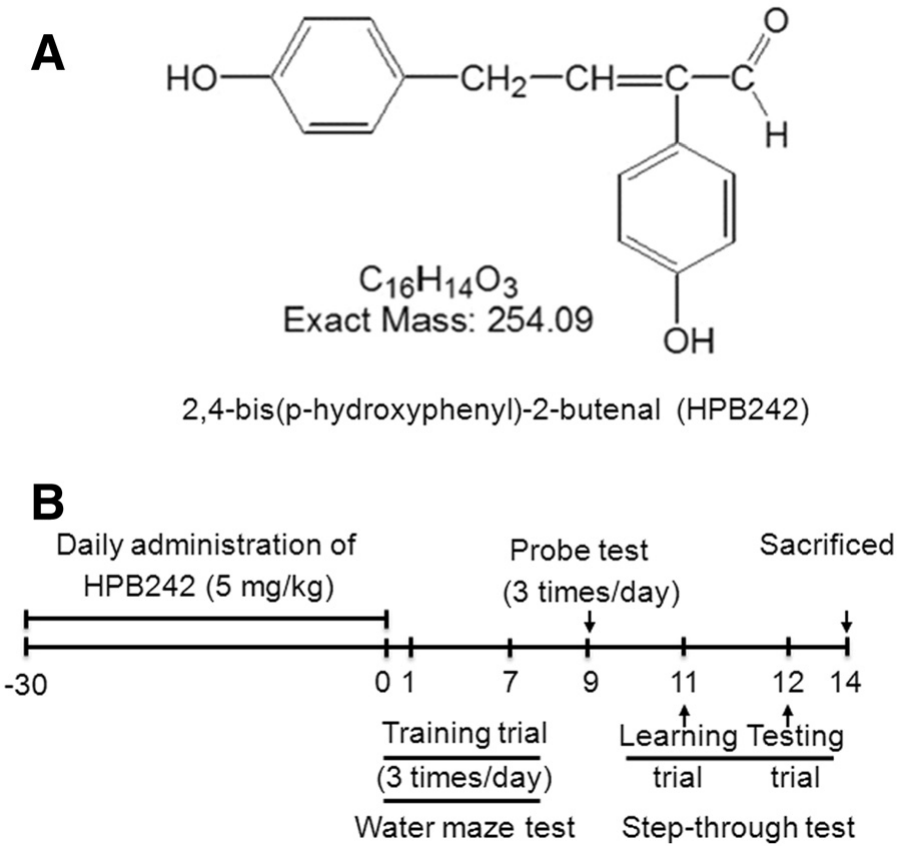
**Structure of HPB242 and the scheme of experimental study on the mice models.** A tyrosine-fructose Maillard reaction product HPB242 (**A**) and scheme for animal treatments and behavioral tests. (**B**) The animals received HPB242 through drinking water (5 mg/kg) for 1 month, and then memory tests were conducted.

Characterization of HPB242 has been described elsewhere [34]. In brief, we prepared 100 ml of fructose-tyrosine mixture including 0.1 M tyrosine and 0.05 M fructose. MR was carried out in temperature-controlled autoclave apparatus (Jisico, Seoul, South Korea) at 130°C for 2 hr. After 2 hr heating, the reaction mixture was filered through a 0.45 μm membrane and used to isolate the active compounds through several fractionation steps. Fructose-tyrosine MR products were purified using a series of solvent fractionations; ethyl acetate (EtOAc), n-butanol, and water. The EtOAc fraction was subjected to silica gel column chromatography and eluted with increasing concentrations of methanol (MeOH) in dichloromethane (DCM). An A2 fraction via DCM:MeOH (20:1, v:v) of a first silica gel chromatography was separated to 23 sub-fractions via a second silica gel chromatography. Fractions B14 and B15 from the 23 sub-fractions were further purified by semi-preparative high performance liquid chromatography with a C18 column. The structure is shown in Figure 1A. Then 2,4-Bis(phydroxyphenyl)-2-butenal was given to Tg2576 mice (n = 8 in each group) in drinking water at the dose of 5 mg/kg for 1 month.(Figure 1B).

### Morris water maze test

The Morris water maze test is a widely accepted method for examining cognitive function and was used in the present study as described previously [35]. Briefly, a circular plastic pool (height 35 cm, diameter 100 cm) was filled with water (plus white dye) maintained at 22 to 25°C. An escape platform (height 14.5 cm, diameter 4.5 cm) was submerged 0.5 to 1 cm below the surface of the water. The test was performed three times a day for 7 days during the acquisition phase (days 1 to 7), with three randomized starting points. The position of the escape platform was kept constant. Each trial lasted for 60 s or ended as soon as the mice reached the submerged platform. The swimming pattern of each mouse was monitored and recorded by a camera mounted above the center of the pool, and the escape latency, escape distance and swimming speed were assessed by the SMART-LD program (Panlab, Barcelona, Spain). A quiet environment, consistent lighting, constant water temperature and a fixed spatial frame were maintained throughout the experimental period.

### Probe test

To assess memory consolidation, a probe test was performed 48 hr after the water maze test (on day 9). For the probe test, the platform was removed from the pool and the mice were allowed to swim freely. The swimming pattern of each mouse was monitored and recorded for 60s using the SMART-LD program (Panlab). Consolidated spatial memory was estimated by the time spent in the target quadrant area.

### Passive avoidance test

The passive avoidance response was determined using step-through apparatus (Med Associates, Georgia, VT, USA). At 48 hr after the probe test (on day 11), a training trial was performed. To this end, each mouse was placed in the illuminated compartment of the apparatus facing away from the dark compartment. When the mouse moved completely into the dark compartment, it received an electric shock (0.45 mA, 3s duration). At 24 hr after the training trial (on day 12), each mouse was placed in the illuminated compartment and the latency period until it entered the dark compartment was determined and defined as the step-through latency. The cut-off time for the examination was 180s.

### Collection and preservation of brain tissues

At 48 hr after the passive avoidance test, mice were anesthetized with diethyl ether and then perfused with PBS. The brains were immediately removed from skull, and the cortex and hippocampus were dissected on ice. All brain tissues were stored at −80°C until biochemical analysis.

### Immunohistochemical staining

After being anesthetized with diethyl ether, subgroups of mice were perfused intracardially with 50 mL saline. The brains were removed from the skull and post-fixed in 4% paraformaldehyde for 24 h at 4°C. The brains were transferred to 30% sucrose solutions. Subsequently, brains were cut into 30 μm sections by using cryostat microtome (Leica CM1850; Leica Microsystems, Seoul, Korea). After multiple washing in PBS, endogenous peroxidase activity was quenched by incubation of the samples in 3% hydrogen peroxide in PBS for 30 minutes, followed by a 10-minutes wash in PBS. Sections were then incubated for 2 h at room temperature with a mouse polyclonal antibody against Aβ (1:5000; Covance, Berkeley, CA, USA), a rabbit polyclonal antibody against GFAP and iNOS (1:1000; Abcam, Inc, Cambridge, MA, USA), a rabbit polyclonal antibody against COX-2 (1:1000; Cayman, Ann Arbor, MI, USA) and a rabbit polyclonal antibody against ionized calcium binding adaptor molecule 1 (Iba1) (1:1000; Wako, Osaka, Japan). After incubation with the primary antibodies, sections were washed in PBS before being incubated for 1 h at room temperature in the presence of biotinylated goat anti-rabbit or anti-mouse IgG secondary antibodies (1:1000; Vector Laboratories, Burlingame, CA, USA). Sections were then washed with PBS and incubated with avidin-peroxidase complex (Vector Laboratories) for 30 minutes before the immunocomplex was visualized using the chromogen 3,3′-diaminobenzidine (Vector Laboratories). Sections were then counterstained with hematoxylin. Finally, sections were dehydrated in ethanol, cleared in xylene and covered with Permount (n = 8 mice per group).

### Immunofluorescence staining

The brain-tissue processing methods were the same as described above (Immunohistochemical staining). The mouse brain sections were incubated for 2 h at room temperature with a goat polyclonal antibody against p50 (1:500, Santa Cruz Biotechnologies, Inc., Santa Cruz, CA, USA), a rabbit monoclonal antibodies against Iba1 (1:1000; Wako, Osaka, Japan) and a rabbit monoclonal antibodies against GFAP (1:1000; Abcam). After washing with PBS, the brain sections were incubated with an anti-rabbit or anti-mouse secondary antibody labeled with Alexa-Fluor 488 and Alexa-Fluor 568 (1:800 Invitrogen, Paisley, UK) for 2 h at room temperature. Sections were then dehydrated in ethanol, cleared in xylene and covered with Permount. Final images were acquired using a confocal laser scanning microscope (TCS SP2, Leica Microsystems AG, Werzlar, Germany).

### Western blot analysis

Western blot analysis was performed with the hippocampus or cortex dissected and stored at −80°C. The brain tissues were homogenized with lysis buffer (PRO-PREP; iNtRON, Sungnam, Korea; n = 8 mice per group) and centrifuged at 2500 × g for 15 minutes at 4°C. Equal amounts of total protein (40 μg) isolated from brain tissues were resolved on 8% or 10% sodium dodecyl sulphate polyacrylamide gels and then transferred to nitrocellulose membranes (Hybond ECL; Amersham Pharmacia Biotech, Piscataway, NJ, USA). Membranes were incubated at room temperature for 2 h with the following specific antibodies: anti-BACE1 (1:500; Sigma, St Louis, MO, USA), anti-APP (1:500; Sigma), anti-iNOS, anti-GFAP (both 1:1000; Abcam), anti-Iba1 (1:1000; Wako), anti-COX-2 (1:1000; Cayman), anti-p50, anti-p65, anti-IκB, anti-p-IκB, anti-STAT1/3, anti-p-STAT1/3 (all diluted 1:500, Santa Cruz Biotechnologies) and anti-β-actin (1:2500; Sigma). Blots were then incubated at room temperature for 2 h with corresponding peroxidase-conjugated anti-mouse or anti-rabbit antibodies (1/2000; Santa Cruz). Immunoreactive proteins were detected using an enhanced chemiluminescence (ECL) western blotting detection system. The relative density of the protein bands was scanned densitometrically using My Image (SLB, Seoul, Korea) and quantified by Lab Works 4.0 (UVP, Upland, CA, USA).

### Gel electromobility shift assay (EMSA)

Gel shift assays were performed according to the manufacturer’s recommendations (Promega, Madison, WI, USA). Briefly, brain tissues (100 mg) were suspended in 200 μl of solution A (1 M HEPES (pH 7.9), 0.15 M MgCl_2_, 1 M KCl, 100 mM dithiothreitol, 0.5% Nonidet P-40, 0.1% Protase Inhibitor (Sigma), 0.1% Phosphatase Inhibitor (Sigma) and 100 mM phenylmethylsulfonyl fluoride). Tissues were then allowed to incubate on ice for 6 minutes and were centrifuged at 8000 rpm for 8 minutes. The pelleted nuclei were resuspended in solution C (solution A + (5 M NaCl, 20 mM ethylenediamine tetraacetic acid anticoagulant (EDTA), 20% glycerol)-(Nonidet P-40, 1 M KCl)) and allowed to incubate on ice for 20 minutes. The tissues were centrifuged at 15000 rpm for 15 minutes, and the resulting nuclear extract supernatant was collected in a chilled Eppendorf tube. Consensus oligonucleotides were endlabeled using T4 polynucleotide kinase and (γ-^32^P) ATP for 10 minutes at 37°C. Gel shift reactions were assembled and allowed to incubate at room temperature for 10 minutes followed by the addition of 1 μL (50,000 to 200,000 rpm) of ^32^P endlabeled oligonucleotide and another 20 minutes of incubation at 37°C. Subsequently 1.5 μL of gel loading buffer was added to each reaction and loaded onto a 6% non-denaturing gel, and electrophoresis was performed until the dye was four fifths of the way down the gel. The gel was dried at 80°C for 2 h and exposed to film overnight at −70°C. The relative density of the protein bands was scanned by densitometry using MyImage (SLB, Seoul, Korea), and quantified by Labworks 4.0 software (UVP Inc.).

### Measurement of Aβ_1–42_

A specific ELISA kit (Immuno-Biological Laboratories, Gunma, Japan) was used to determine Aβ(1 to 42) levels. Protein was extracted from brain tissue using lysis buffer containing protease inhibitors and centrifuged 2500 × g for 15 minutes at 4°C. The supernatant was collected. Briefly, 100 μL aliquots of brain tissue samples (total protein 100 μg) from eight mice in each group were added to pre-coated plates and incubated overnight at 4°C. After washing each well seven times with washing buffer, 100 μL chromogen-labeled antibody solution was added and the mixture was incubated for 1 h at 4°C in the dark. After washing each well nine times with washing buffer, 100 μL chromogen was added and the mixture was incubated for 30 minutes at room temperature in the dark. Finally, after added 100 μL stop solution, the resulting color was assayed at 450 nm using a microplate reader (Sunrise; TECAN, Mannedorf, Switzerland).

### Measurement of β-secretase

β-secretase activity in Tg2576 mice brains was determined using a commercially available β-secretase activity kit (Abcam). Protein was extracted from brain tissue using ice-cold extraction buffer, incubated on ice 20 minutes and centrifuged 10000 × g for 5 minutes at 4°C. The supernatant was collected. A total of 50 μL of sample (total protein 100 μg) was added to each well followed by 50 μL of 2 × reaction buffer and 2 μL of beta-secretase substrate incubated in the dark at 37°C for 2 hr. Fluorescence was read at excitation and emission wavelengths of 355 and 510 nm respectively, using a Fluostar Galaxy fluorimeter (BMG Lab Technologies, Offenburg, Germany) with Felix software (BMG Lab Technologies, Offenburg, Germany). Beta-secretase activity is proportional to the fluorimetric reaction, and is expressed as nmol/mg protein per minute.

### Statistical analysis

All statistical analysis was performed with GraphPad Prism 4 software (Version 4.03; GraphPad software, Inc., San Diego, CA, USA). Group differences in the escape distance, latency, and velocity in the Morris water maze task were analyzed using two-way analysis of variance (ANOVA) with repeated measures, the factors being treatment and testing day. Otherwise, data were analyzed by one-way ANOVA followed by Dunnett’s post hoc test. All values are presented as mean ± standard error of the mean (SEM). Significance was set at *P* < 0.05 for all tests.

## Results

### Inhibition of memory impairment in Tg2576 mice by HPB242

**Figure 2:**
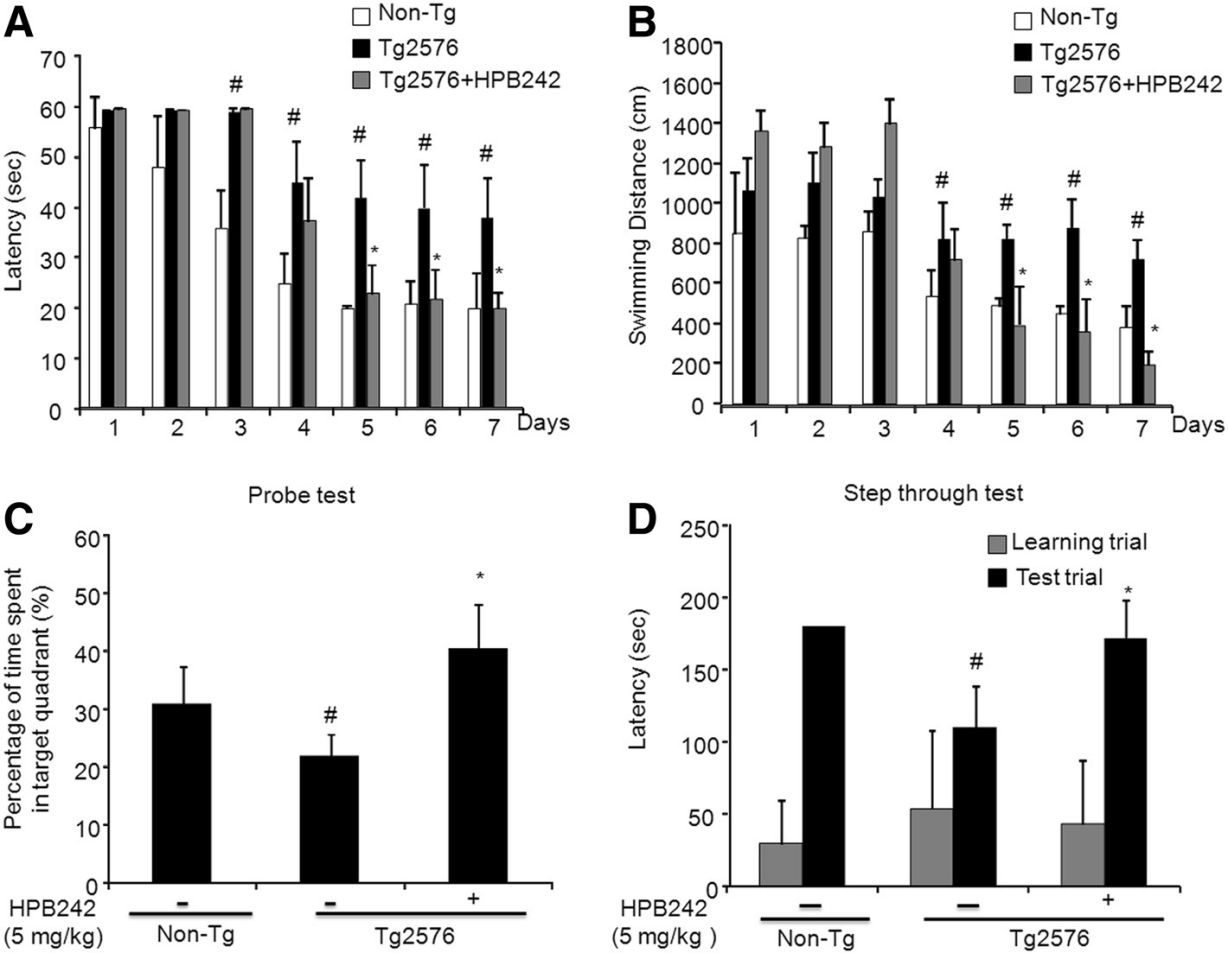
**Effect of HPB242 on improvement of memory impairment in Tg2576 mice.** The training trial was performed three times a day for 7 days. Swimming time (**A**) and swimming distance (**B**) to the platform were automatically recorded. Two days after the training trials, a probe test was performed. The time spent in the target quadrant and target site crossing within 60 s was represented (**C**). Each value is presented as mean ± standard error of the mean (SEM) from eight mice. To perform the passive avoidance test, mice were given an electric shock on entering the dark compartment for training on the learning day. After 2 days, the retention time in the illuminated compartment was recorded (**D**). Each value is presented as mean ± SEM from eight mice. #Significantly different to non-Tg mice (*P* < 0.05), *Significantly different to non-treated Tg2576 mice (*P* < 0.05).

To investigate the preventive effect of HPB242 against memory impairment and Aβ_1-42_ depositions in the AD model mice, we treated 12-month old Tg2576 transgenic mice with HPB242 for 1 month, and then compared memory deficiency with the non-treated mice using the water maze test. The Tg2576 mice were trained for three trials per day for 7 days. Escape latency and escape distances, which are the time and distance travelled to reach the platform in the water maze, were measured to determine the memory-improving effect of HPB242. The mice exhibited shorter time and shorter escape latency with the training, however, the escape latency of Tg2576 mice was not much reduced compared to the non-transgenic mice. Oral treatment with HPB242 (5 mg/kg) for 1 month significantly ameliorated memory dysfunction in the AD model mice. Statistical analysis of data from day 5 showed a significant memory-improving effect of HPB242 treatment. Escape latency (*F* (1, 15) = 9.61, *P* < 0.05 (treatment-wise)) (*F* (6, 15) = 12.83, *P* < 0.05 (day-wise)) of the treated group was shorter than that of the non-treated group (Figure 2A). Escape distance (*F* (1, 15) = 10.31, *P* < 0.05 (treatment-wise); *F* (6, 15) = 5.51, *P* < 0.05 (day-wise)) was also reduced by the treatment (Figure 2B). However, there was no significant difference in average speed between the non-treated and the HPB242-treated group (data not shown).

After the water maze test, we performed a probe test to analyze maintenance of memory. During the probe test, the time spent in the target quadrant by the Tg2576 mice group treated with HPB242 (18.78 ± 4.72 s) was significantly increased compared with the non-treated group (36.87 ± 8.14 s) (*F* (1, 15) = 207.84, *P* < 0.05) (Figure 2C). In particular, the time spent by HPB242-treated Tg2576 mice was similar to the time spent by non-transgenic mice (27.31 ± 10.73s).

We then evaluated learning and memory capacities by the passive avoidance test using the step-through method. In the passive avoidance test, there was no significant difference on the learning trial. However, in the test trial, Tg2576 mice treated with the HPB242 significantly increased the step-through latency (173.33 ± 36.56 s) compared with the non-treated transgenic mice (100.16 ± 32.49 s) (*F* (1, 15) = 11.26, *P* < 0.05) (Figure 2D).

### Effect of HPB242 on Aβ accumulation and amyloidogenesis in brains of Tg2576 AD mice

**Figure 3:**
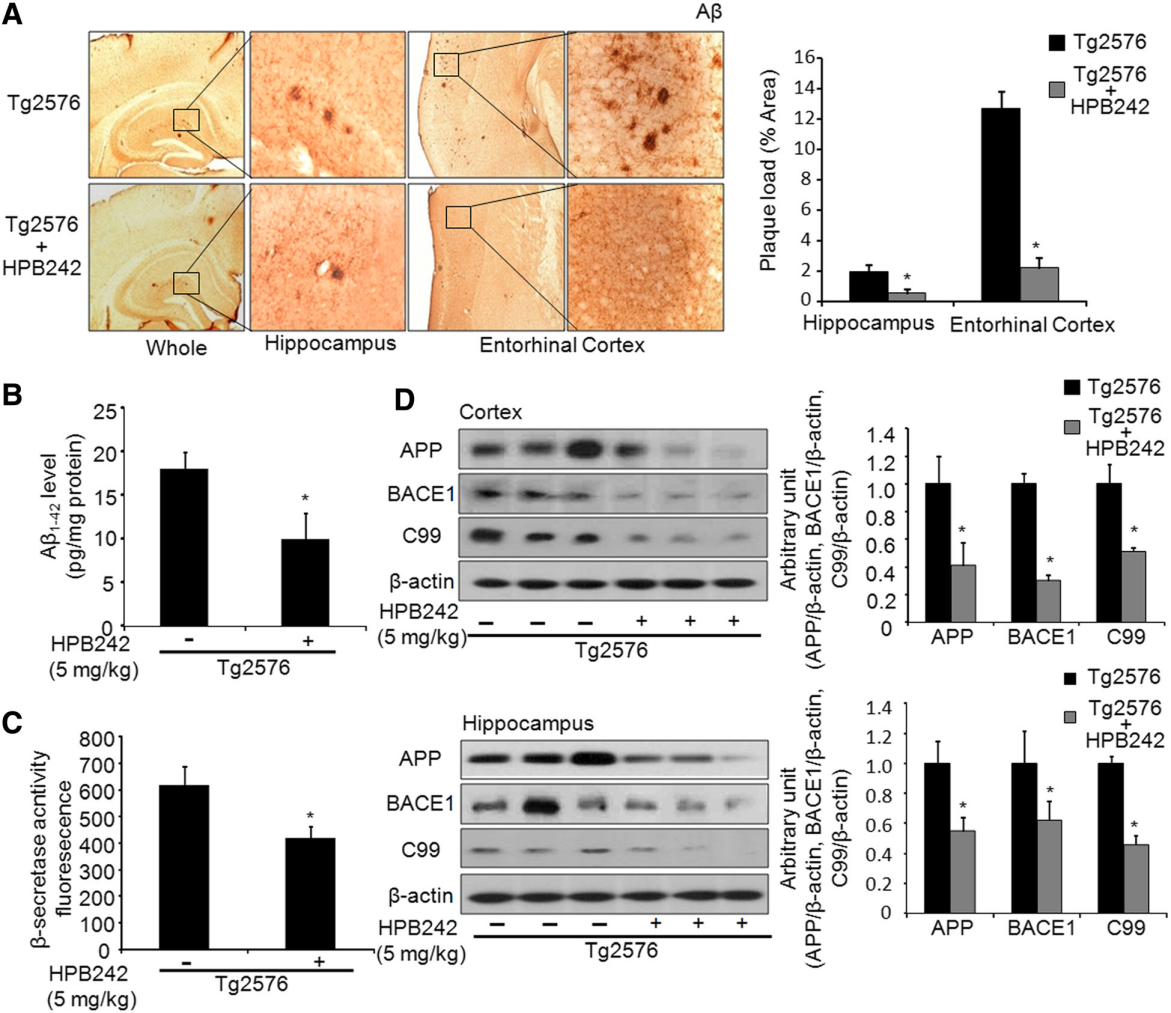
**Inhibitory effects of HPB242 on accumulation of Aβ**_**1-42**_**in the brain of Tg2576 mice.** Aβ accumulation in the brains of Tg2576 mice was determined by immunohistochemical analysis using Aβ_1-42_-specific antibody (**A**). The sections of Tg2576 mouse brains were incubated with anti-Aβ_1-42_ primary antibody, and biotinylated secondary antibody. Immunoperoxidase staining of brains of Tg2576 and treated-Tg2576 mice shown (brown color). Aβ_1-42_ level was measured in mouse brains by ELISA as described in Materials and Methods (**B**). The value is mean ± standard error of the mean (SEM) (n = 8 mice). The activity of β-secretase was investigated using assay kit as described (**C**). Values measured from each group of mice were calibrated by the amount of protein and expressed as mean ± SEM (n = 8 mice). The expression of APP and BACE1 were detected by western blotting using specific antibodies in the mouse brain. Each blot is representative of three experiments (**D**). *Significantly different from non-treated Tg2576 mice (*P* < 0.05).

Several studies reported that Aβ accumulation, which is thought to be a major cause of AD, occurred in the brain of Tg2576 mice. Hence, we investigated whether HPB242 attenuated Aβ accumulation in the brains of Tg2576 mice. Immunohistochemical analyses using Aβ_1–42_-specific antibodies revealed clearly the Aβ deposition in the cortex and hippocampus of non-treated Tg2576 mice. In contrast, there appeared to be reduced Aβ accumulation in the brains of HPB242-treated Tg2576 mice, as evidenced by a decrease in the number of Aβ plaques (Figure 3A). To determine whether the degree of Aβ deposition is paralleled with Aβ protein levels in the brain, quantitative analysis of Aβ_1–42_ levels was performed using an Aβ_1–42_-specific ELISA kit. As shown in Figure 3B, HPB242 treatment reduced Aβ_1–42_ levels in the brain of Tg2576 mice. In addition, to evaluate β-secretase activity (BACE1) we performed ELISA, and western blot analysis was performed to quantify β-secretase activity, APP and BACE1. ELISA analysis revealed that β-secretase activity was significantly reduced by treatment with HPB242 in the brain of Tg2576 mice (Figure 3C). Western blot analysis also revealed that APP, C99 and BACE1 levels were significantly decreased in both the cortex and hippocampus of HPB242-treated mice compared with non-treated mice (Figure 3D).

### Effect of HPB242 on activation of astrocytes and microglia, and expression of iNOS and COX-2 in the Tg2576 mice brain

**Figure 4:**
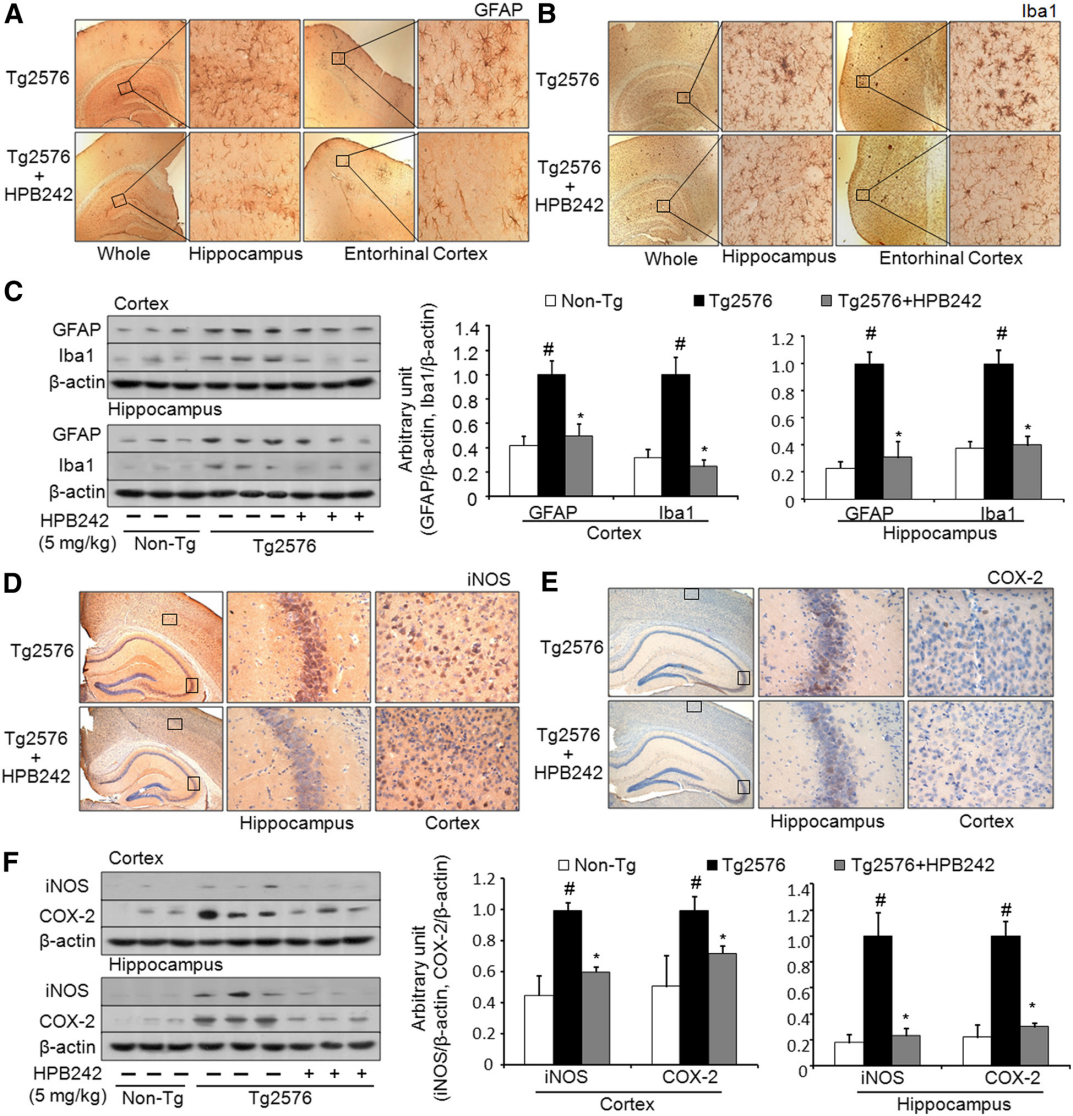
**HPB242 inhibited activation of astrocytes and microglia, and reduced expression of COX-2 and iNOS in the brains of Tg2576 mice.** The effect of HPB242 on reactive astrocytes and activated microglia cells was measured by immunohistochemical analysis and western blotting analysis. The sections of mice brain incubated with anti-glial fibrillary acidic protein (GFAP) (**A**) or ionized calcium binding adaptor molecule 1 (Iba1) (**B**) primary antibody and the biotinylated secondary antibody (n = 8). The stained tissues were viewed with a microscope (×100 or 400). Expression of GFAP and Iba1 were also examined by specific antibodies in the cortex and hippocampus (**C**). Each blot is representative for three experiments (n = 8). Inhibitory effects of HPB242 on the Tg2576 mice brain expression of inflammatory proteins. The sections of mouse brain incubated with anti-iNOS (**D**) or COX-2 (**E**) and the biotinylated secondary antibody (n=8). The resulting tissue was viewed with a microscope (×100 or 400). The expression of inducible nitric oxide synthase (iNOS) and cyclooxygenase-2 (COX-2) were detected by western blotting using specific antibodies (**F**). Each blot is representative for three experiments (n = 8). *Significantly different to non-treated Tg2576 mice (*P* < 0.05).

It has also been reported that activation of astrocytes and microglia is one of the characteristic features of AD; these can produce pro-inflammatory cytokines as well as generate Aβ on activation. We thus examined activation of astrocytes and microglia in the brains of HPB242-treated and non-treated Tg2576 mice. In the results, GFAP-reactive cell number (activated astrocytes) and Iba1-reactive cell number (microglia) was reduced in the brains of HPB242-treated Tg2576 mice compared to those of non-treated Tg2576 mice (Figure 4A and B). To confirm these results, we investigated the expression of GFAP and Iba1 by western blot analysis. The results revealed that GFAP and Iba1 levels were significantly decreased in both the cortex and hippocampus of HPB242-treated compared with non-treated mice (Figure 4C). We also investigated the inhibitory effect of HPB242 on memory impairment and Aβ deposition via inhibition of neuroinflammation; the expression of iNOS and COX-2 were determined by immunohistochemical analysis and western blot. Expression of the inflammatory protein such as iNOS and COX-2 in brain of Tg2576 mice were significantly decreased by treatment of HPB242 (Figure 4D, E and F).

### Effect of HPB242 on NF-κB transcriptional and DNA binding in the brain of Tg2576 mice

**Figure 5:**
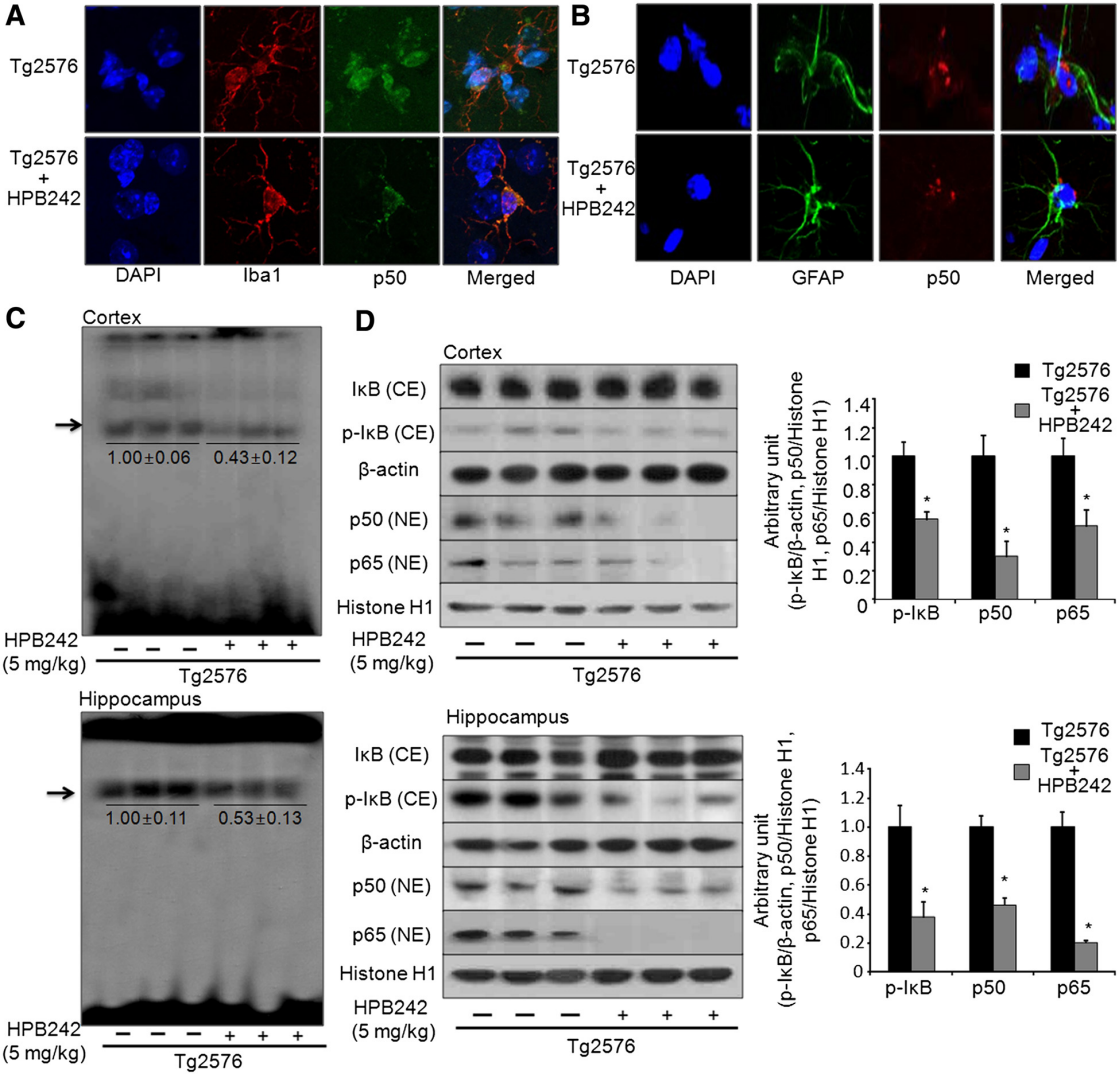
**Inhibitory effect of HPB242 on nuclear factor-kappa B (NF-κB) translocation and DNA binding activity in the Tg2576 mouse brain.** Effect of HPB242 on translocation of p50 in the Tg2576 mice brain; confocal microscopy images of the nuclear translocation of NF-kB-p50 were observed in microglia (**A**) and astrocytes (**B**) in the Tg2576 mouse brain. Effect of HPB242 on NF-κB activity in the Tg2576 mouse brain were determined by gel electromobility shift assay (EMSA) as described in Materials and Methods (**C**). Representative results were obtained from at least three different sets of experiment (n = 8). Inhibitory effect of thiacremonone on nuclear translocation of p50 and p65 and effect of HPB242 on p-IκB and IkB expression in the Tg2576 mice brain (**D**). Nuclear translocation of p50, p65, p-IκB and IκB expression levels in activity in the Tg2576 mice brain were determined by western blot (n = 8).

Since NF-κB is implicated for Aβ generation and neuroinflammatory reaction, we determined NF-κB DNA binding activity and expression of p50 and p65, submits of NF-κB. The analysis of NF-κB translocation on the Tg2576 mouse brain was performed by confocal microscopy. From confocal microscopy analysis, we found that HPB242 prevented NF-κB translocation from cytoplasm to nucleus in the microglia and astrocytes of the Tg2576 mouse brain (Figure 5A and B). Moreover, to confirm this detail, we also investigated the effects of HPB242 on NF-κB activation in Tg2576 mice. This DNAbinding activity of NF-κB confirmed by EMSA analysis was inhibited by HPB242 (Figure 5C). The levels of cytoplasmic phosphorylated IκB, nuclear translocation of p50 and p65 were also inhibited by treatment with HPB242 (Figure 5D).

### Effect of HPB242 on STAT1 and STAT3 activities

**Figure 6:**
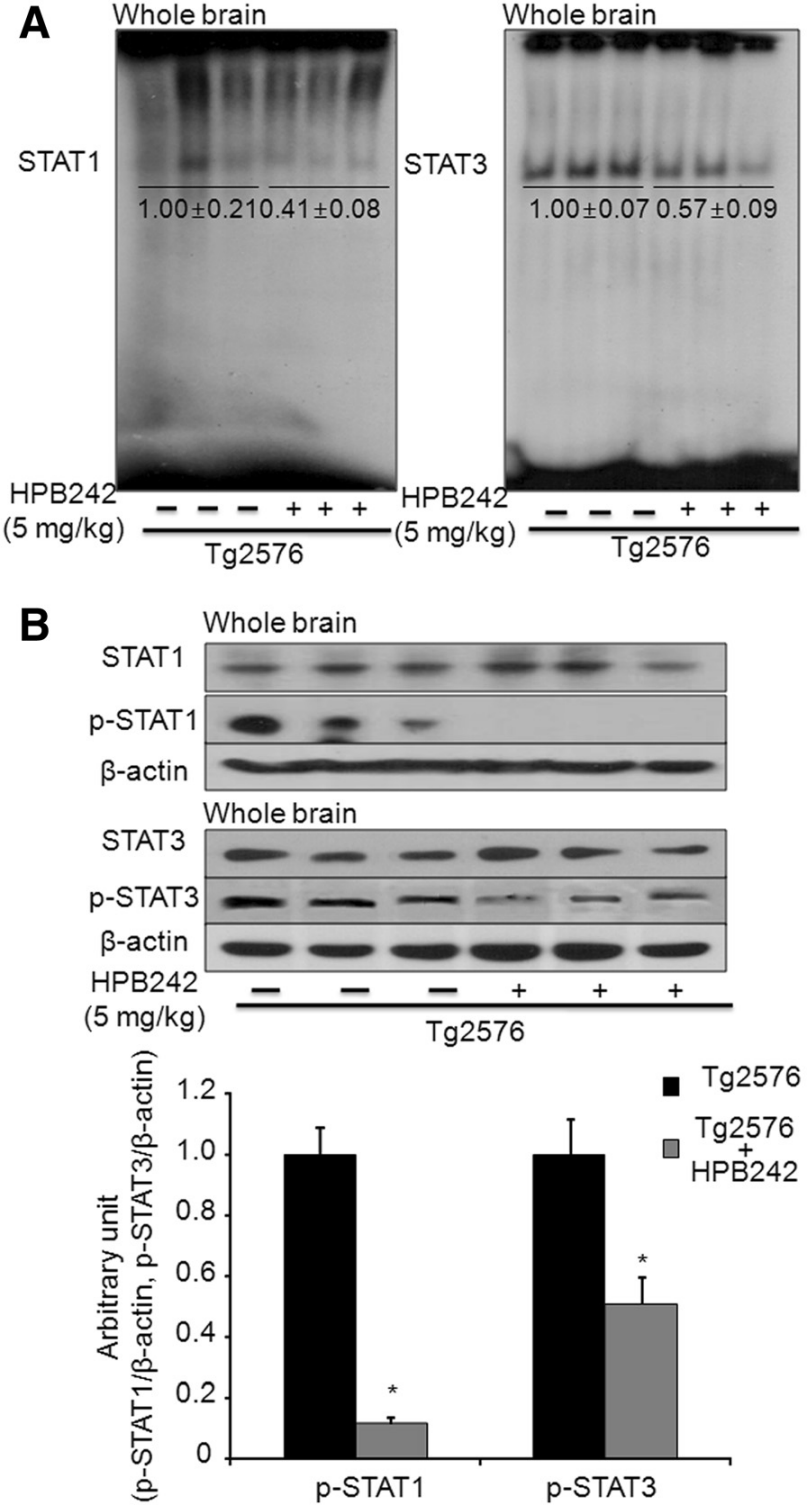
**Effects of HPB242 on signal transducer and activator of transcription (STAT)1 and STAT3 activation in the Tg2576 mice.** Gel electromobility shift assay (EMSA) analysis of STAT1 and STAT3 DNA binding activity in nuclear extracts from the brains (cortex and hippocampus) of HPB242-treated and non-treated Tg2576 mice(**A**). The retarded bands are indicated by an arrow. Representative results were obtained from at least three different sets of experiment (n = 8). The Tg2576 mouse brain extracts were prepared, and phospho-STAT1 and phospho-STAT3 level were detected by western blot (**B**). Each blot was representative of three experiments (n = 8).

In the nucleus, STATs and NF-κB regulate the activity of genes whose products are critical in controlling numerous cellular and organismal processes, including inflammatory responses and Aβ deposition in the brain in. We investigated whether HPB242 can prevent activation of STAT1 and STAT3 in the brain of Tg2576 mice. Our results showed that the DNA bingding ability of STAT1 and STAT3 was inhibited by HPB242 performed by EMSA (Figure 6A). Western blot analysis revealed that p-STAT1 and p-STAT3 levels were significantly decreased in the whole brain of HPB242-treated Tg2576 mice compared with non-treated Tg2576 mice (Figure 6B).

## Discussion

Accumulating epidemiological evidence has suggested that neuroinflammation may contribute to the occurrence and progression of AD [36-38]. The brains of patients with AD appear to display enhancement of hallmarks of neuroinflammation, including marked astrogliosis, and elevated release of proinflammatory mediators and cytokines, as well as microglial activation [6]. Indeed, neuroinflammation has been reported to cause amyloidogenesis in several AD animal models. For example, APP/PS1 is an AD mouse model bearing mutant transgenes of APP and PS1; Aβ deposition and neuroinflammation were present in this mouse model in the early stage of life [39,40]. We also reported that Tg2576 mice displayed Aβ deposition, neuronal dysfunction, neuroinflammation and impairment of spatial memory by overexpression of APP [41]. Furthermore, neuroinflammatory reaction has been detected in Aβ-infused mice [42,43]. It was also reported that several anti-inflammatory compounds such as 4-O-methylhonokiol, thiacremonone and obovatol improved memory functions in AD animal models [6,12,42]. In the present study HPB242 inhibited memory impairment, and suppressed amyloidogenesis via its anti-neuroinflammatory properties in Tg2576 mice. These results indicated that anti-neuroinflammatory effects of HPB242 could be associated with anti-amyloidgenesis, and thus improve memory dysfunction.

BACE1 cleaves APP at the N-terminal position of Aβ [44]. It was reported that the Swedish mutant Tg2576 mice show enhanced cleavage of APP by increase of β-secretase activation [45]. It is known that Aβ is generated from APP by a series of proteolytic processes involving β- and γ-secretases in the amyloidogenic pathway [46]. In the present study, we found that HPB242 reduced Aβ accumulation in Tg2576 mice through inhibition of β-secretase activity and consecutive decrease in expression of APP and C99. Recently, it was also reported that the anti-inflammatory compound, thiacremonone, a sulfur compound isolated from garlic, and 4-O-methylhonokiol effectively inhibited amyloidogenesis through reduced β-secretase activity in LPS-injected mice [12,47]. Activated astrocytes and microglia closely associate to amyloid plaques in AD. They could have a role in the neurotoxicity observed in AD because of the inflammatory reaction they generate [48]. BACE1 is induced in the proximity of activated astrocytes and microglia and upregulated in astrocytes and microglia upon exposure to pro-inflammatory cytokines influencing APP processing and β-secretase activity, to induce amyloidogenesis [49,50]. We found that the number of reactive astrocytes and microglia were elevated in the brain of Tg2576 mice, which was prevented by HPB242. HPB242 also demonstrated anti-inflammatory and anti-amyloidogenic effects in the Tg2576 mouse brain. Similar to these *in vitro* effects of HPB242-like anti-inflammatory and anti-amyloidogenic effects [10], it was also reported that BACE1 transcription is increased by other inflammatory gene expression such as iNOS and COX-2, which are regulated by NF-κB in reactive astrocytes and microglia [51]. In this study, we also found that HPB242 inhibits expression iNOS and COX-2, especially in the hippocampus of Tg2576 mice. Moreover, we found that the reactive cell number for GFAP and Iba1 are also reactive for p50, and the elevated co-reactive cell numbers in the Tg2576 mouse brain was reduced by HPB242. These results suggest that the inhibitory effect of HPB242 on the activation of astrocytes and microglia might be significant to modulate or halt neuroinflammatory-mediated amyloidogenesis.

NF-κB is a positive regulator in the expression of a variety of rapid-response genes involved in inflammatory effects and amyloidogenesis [52]. NF-κB activates the transcription of APP, BACE1 and some of the γ-secretase members and increases protein expressions and enzymatic activities, resulting in enhanced Aβ production [53]. We therefore investigated the activity of NF-κB as a possible mechanism of the anti-inflammatory and anti-amyloidogenesis effect of HPB242. In this study we showed that HPB242 reduced NF-κB activity in the Tg2576 mouse brain. In fact, in the previous *in vitro* studies, we found that HPB242 prevented LPS-induced neuroinflammation and amyloidogenesis in cultured astrocytes and microglia through inactivation of NF-κB [10]. In addition, numerous factors were reported to inhibit amyloidogenesis via suppression of NF-κB such as sorafenib [54], L-theanine [55] and tripchlorolide [56]. Further support comes from a previous study showing that in AD the brain contains increased levels of BACE1, C99 and NF-κB, and NF-κB expression leads to increase of BACE1 promoter activity and BACE1 transcription, while knockout of NF-κB decreases BACE1 gene expression in LPS-injected mice [6]. Moreover, in the present study, we also found that p50 and p65 subunits of NF-κB are translocated into the nucleus of astrocytes and microglia in the brains of Tg2576 mice, and then the translocation of p50 and p65 are inhibited by the treatment of HPB242. It was reported that Aβ activated the NF-κB pathway by selectively inducing the nuclear translocation of the p50 and p65 subunits, and promoted an apoptotic profile of gene expression [19]. Consistent with the effects on amyloidogenesis as well as C99 and BACE1 expression, NF-κB-induced increase in β-secretase activity was also prevented by HPB242. Therefore, inhibiting the effect of NF-κB could be significant in the anti-inflammatory and anti-amyloidogenic effect of HPB242.

STAT1/3 may also be activated in glial cells by a number of cytokines and then translocated from the cytosol to the nucleus [57]. In the nucleus, STATs regulate the activity of genes whose products are critical in controlling β-secretase activity [58]. STAT1/3 is transcriptionally activated by binding to the STAT1/3 binding sequence in the BACE1 promoter region; it is interesting to note that a number of transcription factor binding sites become activated in response to Aβ generation [45]. It has been reported that a humanin (HN) derivative named colivelin completely restored cognitive function in Tg2576 mice by activating the STAT3 [29]. In this study, we showed that HPB242 inhibited phosphorylation of STAT1/3 as well as DNA binding activity. In fact, in the previous studies we also found that blocking STAT3 abolished the inhibitory effect of HPB242 on NF-κB and amyloidogenesis induced by LPS [10]. Additionally, endogenous BACE1 levels were decreased by overexpression of SOCS, an endogenous negative regulator of STAT1 signaling [26], demonstrating that downregulation of STAT1 signaling suppresses BACE1 expression and Aβ generation in neurons [27]. These findings suggest that one mechanism by which HPB242 prevents anti-amyloidogenesis is due to a decrease in phosphorylation of STAT1 and STAT3. In conclusion, our data showed that HPB242 can protect Tg2576 mice from memory impairment through inhibition of NF-κB and STAT1/3, which could result in the inhibition of Aβ_1-42_ accumulation by attenuating β-secretase activity. We suggest that HPB242, a new product from a tyrosine-fructose MR, could be useful for treatment and/or prevention of neuroinflammatory diseases such as AD.

## Abbreviations

AD: Alzheimer’s disease; Aβ: Amyloid-beta; ANOVA: Analysis of variance; APP: Amyloid precursor protein; BACE1: Beta-secretase 1; COX-2: cyclooxygenase-2; DCM: Dichloromethane; ECL: Enhanced chemiluminescence; EDTA: Ethylenediamine tetraacetic acid anticoagulant; EGCG: (−)-epigallocatechin-3-gallate; ELISA: Enzyme-linked immunosorbent assay; EMSA: Gel electromobility shift assay; EtOAcL: Ethyl acetate; GFAP: Glial fibrillary acidic protein; HPB242: 2,4-bis(p-hydroxyphenyl)-2-butenal; Iba1: Ionized calcium binding adaptor molecule 1; IκB: Inhibitor of κB; iNOS: Inducible nitric oxide synthase; LPS: Lipopolysaccharide; MR: Maillard reaction; MeOH: Methanol; NF-κB: Nuclear factor-kappa B; PBS: Phosphate-buffered saline; PS1: Presenilin1; SEM: Standard error of the mean; SOCS: Suppressor of cytokine signaling; STAT1/3: Signal transducer and activator of transcription 1/3.

## Competing interests

The authors declare that they have no competing interests.

## Authors’ contributions

J-TH designed the study and prepared the manuscript. PJ and J-A K performed experiments. H-SJ isolated and characterized 2,4-bis(p-hydroxyphenyl)-2-butenal. D-YC and Y-JL discussed the study. All authors have read and approved the final version of this manuscript.
